# MRI-guided breast biopsy based on diffusion-weighted imaging: a feasibility study

**DOI:** 10.1007/s00330-020-07396-2

**Published:** 2020-10-30

**Authors:** Stefania Montemezzi, Giuseppe Cardano, Silvia Storer, Nicolò Cardobi, Carlo Cavedon, Lucia Camera

**Affiliations:** 1 Radiology Unit, Department of Pathology and Diagnostics, Azienda Ospedaliera Universitaria Integrata – Verona, P.le Stefani 1, 37126 Verona, Italy; 2 Medical Physics Unit, Department of Pathology and Diagnostics, Azienda Ospedaliera Universitaria Integrata – Verona, P.le Stefani 1, 37126 Verona, Italy

**Keywords:** Breast, Magnetic resonance imaging, Biopsy, Diffusion magnetic resonance imaging, Feasibility studies

## Abstract

**Objectives:**

This study evaluated the feasibility of DWI for lesion targeting in MRI-guided breast biopsies. Furthermore, it assessed device positioning on DWI during biopsy procedures.

**Methods:**

A total of 87 biopsy procedures (5/87 bilateral) consecutively performed between March 2019 and June 2020 were retrospectively reviewed: in these procedures, a preliminary DWI sequence (*b* = 1300 s/mm^2^) was acquired to assess lesion detectability. We included 64/87 procedures on lesions detectable at DWI; DWI sequences were added to the standard protocol to localize lesion and biopsy device and to assess the site marker correct positioning.

**Results:**

Mass lesions ranged from 5 to 48 mm, with a mean size of 10.7 mm and a median size of 8 mm. Non-mass lesions ranged from 7 to 90 mm, with a mean size of 33.9 mm and a median size of 31 mm. Positioning of the coaxial system was confirmed on both T1-weighted and DWI sequences. At DWI, the biopsy needle was detectable in 62/64 (96.9%) cases; it was not visible in 2/64 (3.1%) cases. The site marker was always identified using T1-weighted imaging; a final DWI sequence was acquired in 44/64 cases (68.8%). In 42/44 cases (95.5%), the marker was recognizable at DWI.

**Conclusions:**

DWI can be used as a cost-effective, highly reliable technique for targeting both mass and non-mass lesions, with a minimum size of 5 mm, detectable at pre-procedural DWI. DWI is also a feasible technique to localize the biopsy device and to confirm the deployment of the site marker.

**Key Points:**

*• MRI-guided breast biopsy is performed in referral centers by an expert dedicated staff, based on prior MR imaging; contrast agent administration is usually needed for lesion targeting.*

*• DWI represents a feasible, highly reliable technique for lesion targeting, avoiding contrast agent administration.*

*• DWI allows a precise localization of both biopsy needle device and site marker.*

## Introduction

Breast magnetic resonance imaging (MRI) has been increasingly used for breast cancer screening, detection, and staging, according to the European Society of Breast Cancer Specialists (EUSOMA) recommendations [1]. MRI retains a very high sensitivity, reported over 90%, and has the capacity to detect 20% lesions more than mammography and ultrasound in women with recently diagnosed breast cancer [2–5].

However, breast MRI has a limited specificity, mostly reported ranging from 72 to 90%, in discriminating between benign and malignant lesions [2, 4, 6, 7]. To overcome these limitations, diffusion-weighted imaging (DWI) and maps of the apparent diffusion coefficient (ADC) have been added to breast dynamic contrast-enhanced MRI (DCE-MRI) in a multiparametric approach [4, 8–10].

DWI with *b* values > 600 s/mm^2^ has a reported pooled sensitivity of 89% and a pooled specificity of 84% for breast cancer detection, higher than contrast-enhanced breast MRI [11]. A *b* value ≥ 1000 s/mm^2^ improves the discrimination of breast lesions; moreover, combined DWI and DCE-MRI provide a very high diagnostic accuracy, with a reported sensitivity of 92% and specificity of 86% [8].

In absence of a sonographic/mammographic correlate, a suspicious lesion detected at MRI should be investigated through an MRI-guided biopsy [12–15]. Biopsy is recommended for lesions classified as BIRADS 4 or 5 and for BIRADS 3 lesions in high-risk patients [1, 16].

MRI-guided biopsy is a procedure performed in referral centers by an expert dedicated staff after an accurate review of a previous complete diagnostic contrast-enhanced MRI. MRI-guided vacuum-assisted breast biopsy (MRI-VAB) has a reported malignancy detection rate ranging from 25 to 61%, a reported specificity ranging from 92.2 to 100%, a sensitivity from 79.7 to 93%, a positive predictive value (PPV) from 43.1 to 100%, and a negative predictive value (NPV) from 62.7 to 96.6% [17–23]. The wide range in reported values may depend on differences between populations of studied patients, operators, devices, and histopathological examinations.

Commonly used MRI-guided biopsy protocols include high spatial resolution T1-weighted image fat-suppressed (T1-WI FS) gradient-echo (GRE) sequences acquired before and after gadolinium-based contrast agent (GBCA) administration, with subtraction images to precisely localize the lesion in the compressed breast [13]. The rapid contrast washout of the lesion could hinder the biopsy procedure [24], especially in bilateral MRI-guided biopsies performed on the same patient.

In addition, the repeated administration of GBCAs for MRI could be harmful for the patients, due to gadolinium dose-dependent deposition, that is more pronounced with linear GBCAs [25]. For these reasons, in 2017, the U.S. Food and Drug Administration (FDA) began requiring warning labels on all GBCAs [26]. The European Medicines Agency (EMA) confirmed restrictions on the use of linear gadolinium agents and recommended to use macrocyclic GBCAs in the lowest dose effective for diagnosis and only when unenhanced body scans are not suitable as well [27].

The purpose of our study was to establish feasibility of DWI for lesion detection and localization during MRI-guided biopsy, avoiding contrast agent administration. Furthermore, we wanted to assess biopsy device precise positioning at DWI during biopsy procedures.

## Material and methods

The study was approved by the institutional review board of the Hospital.

A total of 87 (5/87 bilateral) biopsies consecutively performed in our institution between March 2019 and June 2020 were retrospectively analyzed.

For all the patients, a previous diagnostic breast MRI was available. Main indications to perform a breast biopsy were suspicious enhancing lesions classified as BI-RADS 4 or 5 at diagnostic MRI, not recognizable at a “second-look” ultrasound or at a second-reading mammography. BIRADS 3 lesions detected in high-risk patients were also included, as recommended by the EUSOMA guidelines [1]. General contraindications were as follows: lesion localization near nipple, too close to chest wall, to skin, or to implant. Informed consent was acquired from all participants included in the study.

### Image acquisition

In our institution, breast MRIs are performed on a 3-T system, using a phased-array 7-channel dedicated breast coil (Philips Achieva, Philips Healthcare).

A patient was scanned in prone position, with the breast compressed in a grid device without skin folds: the grid helped in lesion localization, preventing motion during biopsy [28].

During the MRI-VAB procedure, the imaging protocol was usually shorter than normal diagnostic MRI with the aim to target the previously identified breast abnormality. To reduce fat suppression problems due to magnetic field inhomogeneity, a manual shimming approach was adopted.

### Image protocol

Table 1 summarizes the standard MRI-guided biopsy protocol used: it included a preliminary DWI with *b* = 1300 s/mm^2^ and DCE sequences for lesion localization, T1-WI FS GRE and DWI *b* = 1300 s/mm^2^ for needle position confirmation, T1-WI FS GRE to control adequate sampling, and finally T1-WI FS GRE to ensure adequate clip marker placement. A final DWI *b* = 1300 s/mm^2^ was acquired to better confirm site marker positioning whenever the last confirmation T1-WI was limited by air- and blood-induced artefacts.

**Table 1:** MRI-guided biopsy protocol used in our institution

Sequence	Orientation	Slice thickness (mm)	Matrix size (pixel)	FOV (mm)	TR/TE (ms)	NSA	Acquisition time (min)	Rationale
Localizer	Three planes							Includes a sagittal plane with the fiducial marker in the grid
DWI (*b* = 1300 s/mm^2^)	Axial	3	116 × 143	260 × 341	3771/61	7	4′32″	Assess lesion visibility on DWI
DCE 3D-THRIVE	Axial	1.9	380 × 378	340 × 340	4.8/2.4	1	5′35″	Lesion localization
T1-WI FS GRE	Axial	3	380 × 378	340 × 340	4.8/2.4	1	1′52″	Ensure proper position of the biopsy device
DWI (*b* = 1300 s/mm^2^)	Axial	3	116 × 143	260 × 341	3771/61	7	4′32″	Assess visibility and precise position of the biopsy device
T1-WI FS GRE	Axial	3	380 × 378	340 × 340	4.8/2.4	1	1′52″	Control adequate sampling and post-biopsy changes
T1-WI FS GRE	Axial	3	380 × 378	340 × 340	4.8/2.4	1	1′52″	Ensure adequate clip marker placement
DWI (*b* = 1300 s/mm^2^)	Axial	3	116 × 143	260 × 341	3771/61	7	4′32″	Control metallic clip visibility

*DWI*, diffusion-weighted imaging; *DCE*, dynamic contrast-enhanced; *THRIVE*, T1-weighted high-resolution isotropic volume excitation; *T1-WI FS*, T1-weighted imaging with fat saturation; *GRE*, gradient echo; *FOV*, field of view; *TR*, repetition time; *TE*, echo time; *NSA*, number of signal average

In bilateral procedures, the first biopsy was performed using DCE sequences to target the lesion and three T1-WI FS GRE sequences to respectively confirm needle positioning, control adequate sampling, and ensure clip placement. The contralateral biopsy was performed using a preliminary DWI sequence, two DWI scans to confirm needle positioning and adequate sampling, respectively, and a final T1-WI FS GRE to assess site marker deployment.

### Breast MRI–guided biopsy technique

Lesion localization co-ordinates were calculated by a dedicated computer-assisted diagnostic workstation (DynaCAD, Invivo Corporation). A needle guide was positioned in the chosen grid square.

A local anesthetic (lidocaine) was injected. Sampling was performed using a coaxial 9-gauge Suros ATEC^®^ Breast Biopsy and Excision System (Hologic) with a lateral approach. T1-WI FS GRE and DWI sequences were then performed to confirm the optimal obturator placement. The plastic obturator was then replaced with the VAB device; a minimum of 24 vacuum biopsy specimens were obtained, usually in two samplings (1 sample per o’clock position) with an intermediate and final lavage of the biopsy cavity to minimize the hematoma.

At the end of the procedure, a site marker (TriMark, ATEC, Suros Surgical and Excision Systems, Hologic) was placed. The mean duration of the procedure was 40 min (range 35–45 min).

### Data analysis

Two experienced radiologists retrospectively analyzed in consensus diagnostic MRI, biopsy images, and data. Lesion detection was first assessed on DWI and confirmed on DCE subtracted images. Lesion enhancement pattern was classified into three categories (“mass”, “non-mass”, and “focus”). Percentages of mass and non-mass lesions were calculated.

In order to assess feasibility of DWI for lesion localization, the correct position of the biopsy device was verified firstly with DWI, then with T1-WI FS GRE.

Final control of the correct sampling and adequate placement of the site marker were assessed with T1-WI FS GRE as the presence of local signal void in the biopsy chamber; when available, it was confirmed at final DWI. The evaluation sometimes was difficult due to post-biopsy changes.

One patient was preliminarily excluded from the series for an extremely superficially located lesion that did not allow adequate sampling.

A total of 11/87 procedures were excluded because lesions did not show any focal hyperintensity at pre-procedural DWI: these biopsies were performed using DCE sequences to detect and localize lesions and T1-WI FS GRE to control the correct positioning of both the sampling device and the site marker.

A total of 12/87 (2 bilateral) procedures were also excluded because a DWI confirmation after needle positioning was not acquired: the reasons were patient pain and discomfort caused by the prolonged prone position or the lack of time due to the high workload. In particular, 2 bilateral procedures were interrupted due to scanner technical problems and the contralateral biopsy was postponed; the second procedure was then performed on another day using DCE sequences only to save time.

A total of 64/87 (3 bilateral) MRI-guided biopsies performed on lesions that showed hyperintensity at preliminary *b* = 1300 s/mm^2^ sequence were included: in these procedures, needle positioning DWI confirmation sequences were acquired.

Histological results were recorded for each patient.

## Results

Table 2 reports patient details, lesion type and size, needle and marker visibility at DWI, and histological results.

**Table 2:** Patient details, lesion type and size, needle and marker visibility at DWI, and histological results

Patient	Age	Lesion type M, mass; NM, non-mass	Size (mm)	Needle visible on DWI? N, no; Y, yes	Site marker visible on DWI? N, no; Y, yes; NP, DWI not performed	Histopathological classification (B)	Histopathological result
1	83	M	13	Y	Y	5	DCIS G2
2	79	NM	32	Y	NP	5	DCIS G3
3	58	M	6	Y	Y	2	UDH
4	70	M	5	Y	Y	3	ADH
5	66	NM	16	Y	NP	3	ADH
6	72	NM	34	Y	Y	5	DCIS G2
7	55	NM	45	Y	NP	5	DCIS G2
8	60	NM	25	Y	NP	2	UDH
9	67	M	10	Y	NP	2	UDH
10	71	M	7	Y	NP	5	IDC G2
11	71	M	13	Y	NP	5	IDC G2
12	60	NM	30	Y	Y	5	DCIS G3
13	57	NM	20	Y	Y	3	ADH
14	50	M	28	Y	NP	3	UDH, LIN 2
15	45	M	5	Y	NP	2	Fibroadenoma
16	36	NM	43	Y	NP	2	UDH
*17	44	M	10	Y	NP	5	IDC G3
18	52	NM	57	Y	Y	3	ADH
19	43	NM	25	Y	NP	2	UDH
20	58	M	7	N	NP	5	IDC
21	58	M	5	Y	NP	3	Radial scar
22	57	M	5	Y	NP	2	Fibroadenoma
23	54	M	8	Y	NP	2	Adenosis, UDH
24	37	NM	20	Y	Y	2	Adenosis, UDH
25	65	M	8	Y	Y	3	ADH, ALH
26	42	NM	90	Y	Y	2	Adenosis
*27	47	M	8	Y	Y	2	Fibroadenoma
28	58	NM	7	Y	NP	2	Fibroadenoma
29	38	NM	20	Y	NP	2	Fibrosis, UDH
30	50	NM	20	Y	Y	3	FEA, UDH
31	50	NM	25	Y	Y	3	Papillary, UDH
*32	50	M	48	Y	Y	3	Papillary, UDH
33	54	NM	70	Y	Y	2	Adenosis, UDH
34	51	NM	40	Y	NP	5	Pleomorphic LIN
35	75	M	8	Y	Y	2	UDH
36	50	NM	33	Y	Y	2	Adenosis, UDH
37	78	NM	15	N	N	5	IDC, ILC
38	44	M	7	Y	N	2	Stromal fibrosis
39	52	NM	55	Y	Y	5	DCIS G2
40	54	NM	25	Y	Y	3	Papillary, UDH
41	57	M	9	Y	Y	5	DCIS
42	62	NM	10	Y	Y	2	Adenosis
43	47	M	12	Y	Y	5	DCIS G2
44	51	NM	66	Y	Y	5	DCIS G3
45	51	M	8	Y	Y	5	IDC
46	56	NM	67	Y	NP	5	DCIS G3; LIN 2
47	60	M	11	Y	Y	2	UDH
48	40	NM	31	Y	Y	2	UDH
49	78	NM	7	Y	Y	2	Adenosis, UDH
50	32	NM	15	Y	Y	3	Papillary
51	40	NM	40	Y	Y	3	FEA
52	61	NM	33	Y	Y	2	GM
53	49	NM	15	Y	Y	5	DCIS G3
54	62	NM	20	Y	Y	5	IDC
55	51	NM	7	Y	Y	3	LIN 2, ADH
56	57	NM	42	Y	Y	2	UDH
57	40	NM	30	Y	Y	3	LIN 1
58	49	NM	15	Y	Y	3	FEA
59	61	M	9	Y	Y	3	Phyllodes
60	51	NM	7	Y	Y	2	UDH
61	57	M	7	Y	Y	2	Fibroadenoma
62	51	NM	15	Y	Y	3	Radial scar
63	26	NM	21	Y	Y	1	Normal breast
64	48	NM	30	Y	Y	2	Fibroadenoma

*IDC*, invasive ductal carcinoma; *DCIS*, ductal carcinoma in situ; *ILC*, invasive lobular carcinoma; *LIN*, lobular intraepithelial neoplasia; *ADH*, atypical ductal hyperplasia; *ALH*, atypical lobular hyperplasia; *UDH*, usual ductal hyperplasia; *FEA*, flat epithelial atypia; *GM*, granulomatous mastitis

*Bilateral biopsy procedure

No major complications occurred during or shortly after the procedure (0%). The mean age of the patients was 54.7 years (range 26–83 years). All the biopsies were technically successful: the success of the procedure was testified from the radio-pathological agreement at multidisciplinary team meeting.

**Fig. 1:**
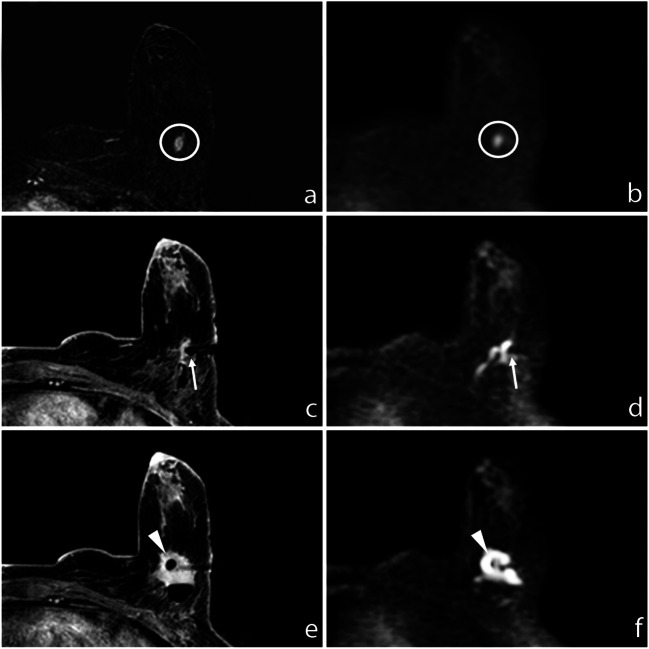
**a** Subtracted DCE 3D-THRIVE (T1-weighted high-resolution isotropic volume excitation) showing a mass lesion (circle) in the deep equatorial plane of the left breast of a 75-year-old woman. **b** DWI (*b* = 1300 s/mm^2^) showing hyperintensity of the enhancing lesion (circle). **c** T1-WI FS GRE showing the proper position of the biopsy device (arrow); the end of the needle is recognizable at (**d**) DWI (*b* = 1300 s/mm^2^, arrow). **e** T1-WI FS GRE showing the correct deployment of the site marker that is also recognizable at (**f**) final DWI (*b* = 1300 s/mm^2^, arrowhead)

The size range of mass lesions measured on post-contrast subtracted T1-WI DCE was between 5 and 48 mm, with a mean size of 10.7 mm and a median size of 8 mm. Non-mass lesions size ranged between 7 and 90 mm, with a mean size of 30.4 mm and a median size of 25 mm. The mean size of lesions not detectable at DWI was 18.8 mm; the median size was 15 mm (Fig. 1).

In 3 cases, a bilateral biopsy was performed: in these patients, DCE sequences were used to target the lesion on one breast, while the contralateral lesion was successfully detected and targeted only by DWI, avoiding an unnecessary new administration of contrast agent.

**Fig. 2:**
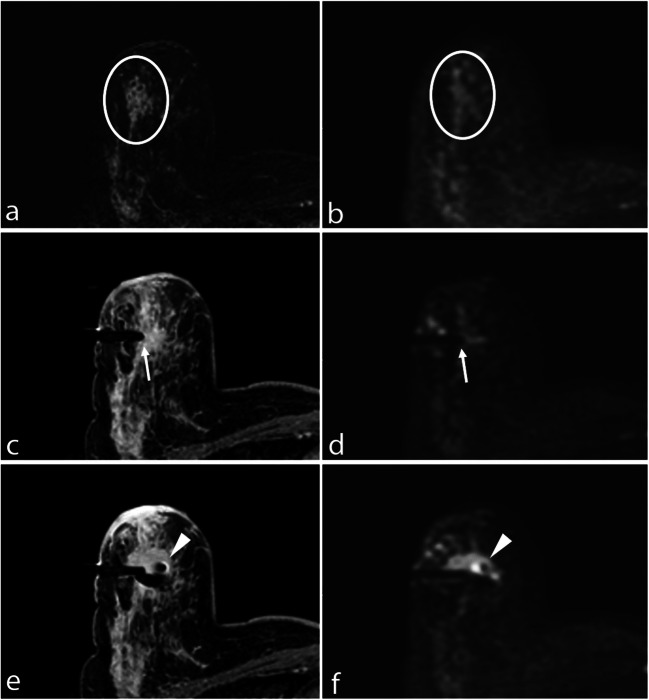
**a** Subtracted DCE 3D-THRIVE showing a non-mass lesion in the upper outer quadrant of the right breast of a 51-year-old woman (circle). **b** DWI (*b* = 1300 s/mm^2^) showing hyperintensity of the enhancing lesion (circle). **c** T1-WI FS GRE showing the proper position of the biopsy device (arrow); the needle is well recognizable at (**d**) DWI (*b* = 1300 s/mm^2^, arrow). **e** T1-WI FS GRE showing the correct deployment of the site marker that is also recognizable at (**f**) final DWI (*b* = 1300 s/mm^2^, arrowhead)

A total of 24/64 (37.5%) lesions were mass and 40/64 (62.5%) were non-mass. Nineteen out of 64 (29.7%) lesions were malignant at histopathological results (B5), 1 was classified as B1 (1.6%), 26 as B2 (40.7%), and 18 as B3 (28%) (Fig. 2).

DWI was acquired to depict the biopsy device position in all the cases included. The biopsy needle was detectable at DWI in 62/64 (96.9%) cases; in few cases, the device was visible only in the terminal part; however, the needle portion with biopsy aperture was always recognizable. DWI evaluation of the device was not successful in 2/64 (3.1%) cases: in one case, it was limited by anesthetic-related artefacts, and in the second case due to deep pre-pectoral localization of the lesion.

The site marker was always correctly identified using T1-WI FS GRE as a focal low signal void; a confirmation DWI was acquired in 44/64 cases (68.8%). In these cases, post-biopsy changes (hematoma, air introduced) limited the identification of the marker at T1-WI; the final DWI sequence helped to better recognize the clip in 42/44 cases (95.5%). In 2/44 cases (4.5%), the marker was not visible at DWI, due to air-induced artefacts. The last DWI was not performed in 20/64 biopsies in order to shorten the procedure: in these procedures, the site marker was correctly identified at T1-WI.

## Discussion

**Fig. 3:**
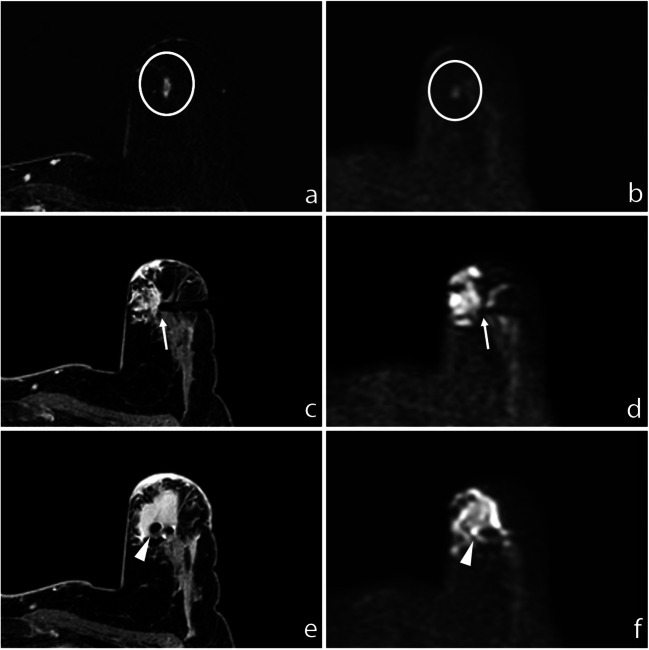
**a** Subtracted DCE 3D-THRIVE showing a mass lesion in the upper inner quadrant of the left breast of a 48-year-old woman (circle). **b** DWI (*b* = 1300 s/mm^2^) showing hyperintensity of the enhancing lesion (circle). **c** T1-WI FS GRE showing the proper position of the biopsy device (arrow); the needle is well recognizable at (**d**) DWI (*b* = 1300 s/mm^2^, arrow). **e** T1-WI FS GRE showing the correct deployment of the site marker that is also recognizable at (**f**) final DWI (*b* = 1300 s/mm^2^, arrowhead)

This study confirmed what previously was observed by Berger et al [29] who showed that DWI may represent a feasible technique for breast lesion detection and targeting in MRI-guided biopsies, avoiding contrast agent administration with the aim to reduce GBCA usage and perform cost-effective procedures. DWI provides a precise localization of both lesion and biopsy device, also in patients with severely impaired renal function at risk of nephrogenic systemic fibrosis or with contrast media allergy that could not undergo a classic DCE-based procedure. Moreover, since DCE sequences are subject to contrast washout and based on GRE imaging, they are prone to motion or respiratory artefacts. Consequently, repeating the sequence or repositioning the patient often represents an issue in DCE-guided procedures: the contrast medium vanishes out and the lesion is not easily detectable anymore. Conversely, EPI (echo planar imaging)-based DWI is highly reliable: the EPI technique allows a fast acquisition compared to dynamic sequences, minimizing motion effects thanks to the high number of signal averages (NSA), with a high signal-to-noise ratio (SNR). Unlike DCE, DWI could be easily repeated after any patient or biopsy device repositioning and is not subject to washout. Moreover, DWI obviates the necessity of contrast agent administration, allowing a significant cost saving (Fig. 3).

Our study demonstrated that DWI also allows lesion localization in bilateral procedures. In fact, in 3 cases, we performed biopsies of two suspected enhancing lesions in both breasts: one lesion was targeted with the DCE sequences, with T1-WI confirmations after needle positioning, sampling, and site marker deployment; the contralateral lesion was successfully detected and targeted only by DWI, avoiding a double contrast agent administration, with a final T1-WI sequence after clip positioning.

In order to plan a DWI-guided breast biopsy, it is mandatory to have a previous, high-quality, diagnostic complete MRI study to assess lesion detectability at high *b* value DWI. If the lesion does not show DWI hyperintensity, the biopsy should be therefore performed with the standard DCE-guided protocol without DWI to shorten the procedure. If the lesion is detectable at high *b* value DWI, *b* = 1300 s/mm^2^ targeting and positioning confirmation sequences can be used to have an optimal contrast between the lesion, the needle device, and adjacent tissues, avoiding contrast agent administration. A *b* = 1300 s/mm^2^ sequence has to be acquired at the beginning of the procedure to confirm lesion detectability: our study demonstrated that DWI can be used for targeting both mass and non-mass lesions. The minimum size of the lesions detectable at *b* = 1300 s/mm^2^ was 5 mm; some lesions were not recognizable because they were located in breasts with abundant fatty component, which hinders diffusion sequences. Based on our experience, to obviate small lesion overlooking, a *b* = 800 s/mm^2^ sequence could be included in the protocol to increase lesion detectability.

**Fig. 4:**
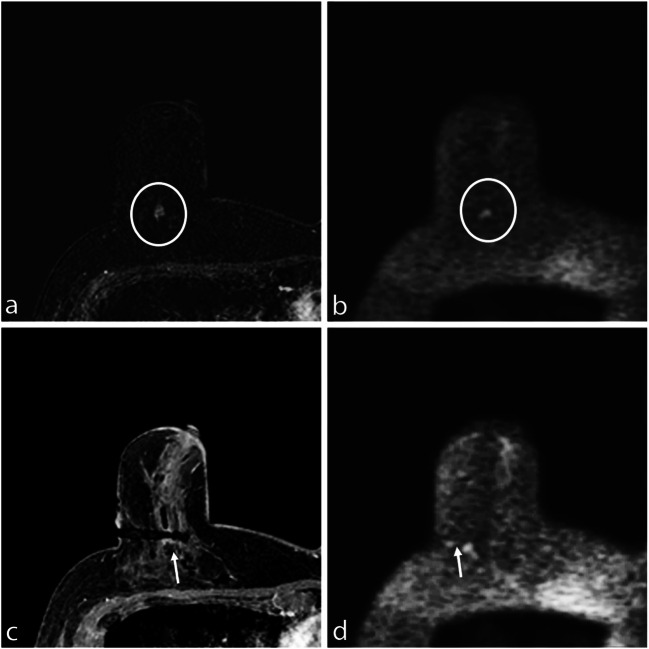
**a** Subtracted DCE 3D-THRIVE showing a mass lesion in the deep equatorial plane of the right breast of a 58-year-old woman (circle). **b** DWI (*b* = 1300 s/mm^2^) showing hyperintensity of the enhancing lesion (circle). **c** T1-WI FS GRE showing the proper position of the biopsy device (arrow); the needle is poorly recognizable at (**d**) DWI (*b* = 1300 s/mm^2^) due to the deep position of the lesion and local anesthetic injection–related artefacts

DWI has proven to be feasible to show the biopsy device position. In our MRI biopsy protocol, the position of device was verified both by T1-WI sequence and DWI: the coaxial system was visible on DWI in 96.9% of cases (62/64). The device was not properly visualized on DWI only in 2/64 cases: one because of artefacts due to anesthetic injection and the other case because of air-tissue interfaces in a deep pre-pectoral lesion (Fig. 4). In fact, EPI-based DWI is prone to artefacts, particularly at higher fields, due to sensitivity to magnetic field inhomogeneity, to air-tissue interfaces, or to inhomogeneous fat suppression [30], that is often related to poor shimming and causes chemical shift artefacts that can hide the biopsy target area. Moreover, injection of anesthetics and small amounts of air increase local magnetic susceptibility differences and static field inhomogeneities, resulting in image degradation. Further distortions are also caused by eddy-currents in the direction of diffusion gradients [30]. Some solutions to these sequence-related issues have been described: optimized radiofrequency coil design, improved shimming techniques, parallel imaging, post-processing [31–33]. In our study, during MRI-guided biopsy, only one *b* value was acquired as a pure detection imaging; thus, the misregistration problem between *b* values due to motion and eddy-currents does not exist. Moreover, to optimize fat suppression, a manual shimming approach was adopted. Thus, these artefacts effectively interfered with the needle precise identification only in 2/64 procedures: when the device was not fully recognizable at DWI, the comparison with T1-WI FS was useful.

After a DWI-guided biopsy, the adequacy check of the sampling should therefore be performed with DWI: although it has a longer duration (4′32″ versus 1′52″), DWI allows a control with better quality compared to fast T1-WI sequences.

**Fig. 5:**
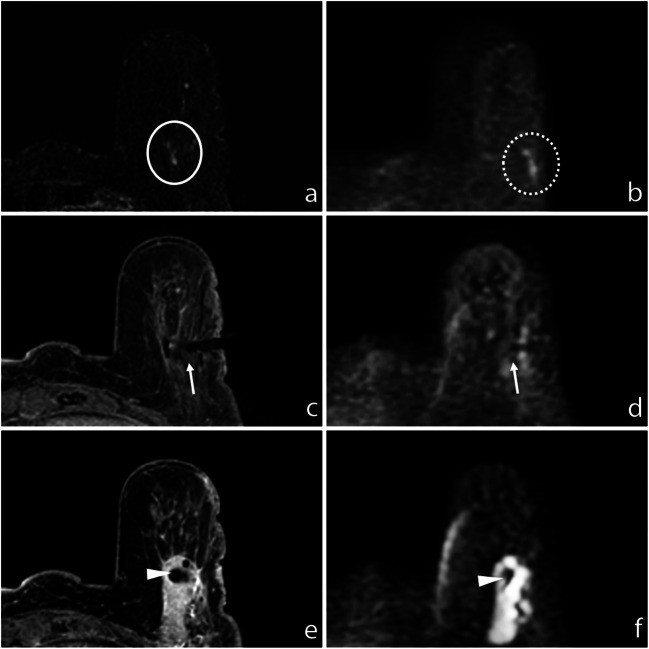
**a** Subtracted DCE 3D-THRIVE showing a non-mass lesion in the inferior sagittal plane of the left breast of a 71-year-old woman (circle). **b** DWI (*b* = 1300 s/mm^2^) showing hyperintensity of the enhancing lesion, slightly lateral-sided than real location due to spatial distortion artefacts (dotted circle). **c** T1-WI FS GRE showing the proper position of the biopsy device (arrow); the end of needle is well recognizable at (**d**) DWI (*b* = 1300 s/mm^2^, arrow). **e** T1-WI FS GRE showing the correct deployment of the site marker (arrowhead) that is also recognizable at (**f**) final DWI (*b* = 1300 s/mm^2^, arrowhead)

Correct site marker deployment has always to be confirmed with T1-WI FS GRE. On the other hand, when post-biopsy changes (hematoma, air introduced by the biopsy) limit the identification of the marker clip at T1-WI FS GRE, a DWI confirmation could help, allowing a better final check, but would lengthen the procedure. In our experience, the T1-WI sequence confirmed the proper position of the clip marker in all cases. A final DWI sequence was also acquired in 44/64 cases (68.8%): in 42/44 (95.5%), the marker was recognizable at DWI; in 2/44 (4.5%), it was not correctly visible, due to air-induced artefacts (Fig. 5).

This study has some limitations: firstly, it is a retrospective study on a relatively small number of patients. In addition, in 20/64 cases, final DWI confirmation was not acquired to save time because of patient discomfort and/or high workload: in our experience, this does not represent a real limitation, because the site marker was always correctly identified using T1-WI FS GRE that is a faster sequence compared to DWI. In fact, the role of the final DWI was only to test its reliability in confirming the correct clip deployment.

DWI sequence time was 4′32″, a duration that might limit feasibility to compliant patients only; to overcome this limitation, we have recently adopted an optimized DWI *b* = 1300 s/mm^2^ that has the same efficacy in lesion detection and targeting, but shorter duration (2′46″ versus 4′32″). The duration of the DWI-guided biopsy protocol we currently use is 10′10″. This topic will be the subject of a future study.

**Fig. 6:**
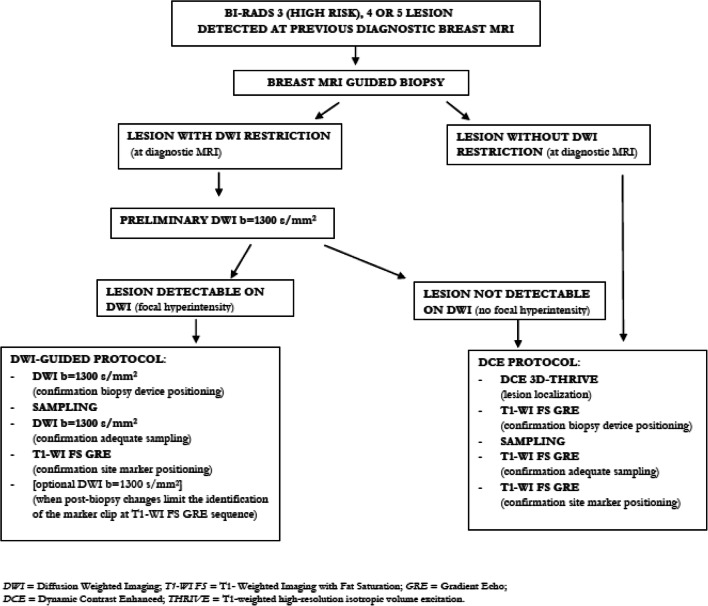
Biopsy protocol flow chart. DWI, diffusion-weighted imaging; T1-WI FS, T1-weighted imaging with fat saturation; GRE, gradient echo; DCE, dynamic contrast enhanced; THRIVE, T1-weighted high-resolution isotropic volume excitation

Figure 6 summarizes our protocol flow chart.

Moreover, this was a feasibility study: the results are promising, but more studies are needed to compare the accuracy of DWI-targeted biopsy to standard protocols with contrast agent administration.

Furthermore, most lesions were non-mass with a large size: this could explain the easy detectability on DWI, whereas this type of enhancing lesions is often overlooked on DWI [34]. To obviate smaller lesion overlooking at DWI *b* = 1300 s/mm^2^, we plan to introduce *b* = 800 s/mm^2^ to increase detectability: in this case, revision of the previous diagnostic MRI should be done to discriminate biopsy target lesion from possible benign lesions with T2 shine-through effects.

In conclusion, in MRI-guided breast biopsy, DWI can be used as a feasible, cost-effective, highly reliable technique for targeting both mass and non-mass lesions, with a minimum size of 5 mm, detectable at pre-procedural DWI. DWI is also a feasible technique to correctly visualize the position of the needle biopsy device throughout the procedure.

The most important advantage of DWI for lesion localization is no need for contrast media administration, a great benefit for patients allergic to gadolinium or presenting renal insufficiency. Moreover, DWI sequences can be easily repeated after any repositioning of the patient or biopsy device and allow localizing the lesion and checking the adequate sampling without any washout issue: these are important advantages over DCE sequences. DWI also allows contralateral breast lesion targeting in bilateral biopsy procedures, regardless of contrast media washout.

The main limitation to a DWI-only-guided biopsy procedure is that EPI-based DWI is partially prone to artefacts due to air, blood, anesthetic injection or air-tissue interfaces. For non-mass lesions, even the small size can be an issue, since they are more difficult to recognize in DWI.

## Abbreviations

DCIS: Ductal carcinoma in situ
EPI: Echo planar imaging
EUSOMA: European Society of Breast Cancer Specialists
FDA: Food and Drug Administration
FS: Fat-suppressed
GBCA: Gadolinium-based contrast agent
GRE: Gradient echo
IDC: Invasive ductal carcinoma
NSA: Number of signal averages
SNR: Signal-to-noise ratio
THRIVE: T1-weighted high-resolution isotropic volume excitation
VAB: Vacuum-assisted biopsy
WI: Weighted image
